# Pain experience in children with juvenile idiopathic arthritis treated with anti-TNF agents compared to non-biologic standard treatment

**DOI:** 10.1186/1546-0096-11-21

**Published:** 2013-05-06

**Authors:** Johanne Jeppesen Lomholt, Mikael Thastum, Troels Herlin

**Affiliations:** 1 Department of Psychology, Aarhus University, Bartholins Allé 9, Aarhus 8000, Denmark; 2 Department of Pediatrics, Aarhus University Hospital, Brendstrupgaardsvej 100, Aarhus N 8200, Denmark

**Keywords:** Juvenile idiopathic arthritis, Pain assessment, Pain management, Anti-TNF agents, DMARDs, Coping, Beliefs

## Abstract

**Background:**

Anti-TNF agents have proven efficacy in children with severe juvenile idiopathic arthritis (JIA) who are unresponsive to standard therapy. Therefore pain reduction or elimination could be expected. The aim of this study was to compare the pain experience in children with JIA treated with anti-TNF agents (n = 41) or non-biologic standard treatment (n = 50).

**Methods:**

All children completed a 2-week pain diary and, for children treated with anti-TNF agents, measures of pain-coping and pain-specific beliefs. Parents rated the child’s level of functional disability. Clinical data were collected from the pediatric rheumatologists.

**Results:**

No significant differences were found between the anti-TNF group and non-biologic standard treatment group for average pain score, number of children with daily pain reported in the pain diary, or level of functional disability. Significantly more children in the anti-TNF group reported no pain at all. Children undergoing standard treatment had significantly higher disease activity. Significant differences were found between the high pain patients treated with anti-TNF agents and the rest of the anti-TNF group in regards to their pain-specific beliefs of disability and harm, and the pain-coping strategy of catastrophizing.

**Conclusion:**

These results indicate that a great proportion of children treated with anti-TNF agents respond well to the treatment in regards to disease activity and pain, but pain was still a problem for a subgroup of children though they were in remission with biological agents. More focus on pain management is needed.

## Background

Pain in children with juvenile idiopathic arthritis (JIA) is more prevalent than previously recognized and with a high daily prevalence [1-3]. Low-dose methotrexate, a disease-modifying antirheumatic drug (DMARD), is considered a first line treatment after the failure of nonsteroidal anti-inflammatory drugs (NSAIDs) and provides some improvement in 60-70% of patients [4-6]. With the advent of biological anti-tumor necrosis factor (anti-TNF) agents the treatment options for JIA have improved markedly. The anti-TNF inhibitors that are currently available have been reported to be efficacious in controlled studies [7-12]. Reducing pain is one of the major treatment goals, but only a few studies on the effect of anti-TNF agents have reported pain as an outcome measure, and those that do use only a single pain measure [7,11-14]. Although anti-TNF agents do reduce and eliminate disease activity and pain in many patients some patients still experience incomplete pain control [15].

In addition to disease activity, multiple factors contribute to the pain experienced by children with JIA [16-20]. Possible mediators of the pain experience in children are pain-coping strategies and pain-specific beliefs [21-23]. Coping is thought to be influenced by a person’s beliefs about a stressor such as pain. A person’s beliefs are defined as “assumptions about reality which serve as a perceptual lens, or a ‘set’ through which events are interpreted” [24]. Primarily coping, but recently beliefs, has been investigated in children with arthritis [17,21-23,25,26].

In our previous studies, we discovered that a high-pain group consisting of children with a greater pain experience and lower disease activity compared to the median values of the total group of patients differed significantly from the remaining sample [21-23]. The high-pain group tended to use the pain-coping strategies of distraction and positive self-statements less and catastrophizing more than the remaining sample of JIA children, and they believed themselves to be more functionally impaired, that pain signifies danger, and that they have less control over their pain compared to the remaining sample [21,22]. For the beliefs of harm and disability the differences were consistent over a 2-year period [23].

### Aim

Few studies have examined pain experience in children treated with anti-TNF agents and only with a single-pain measure [7,11-14]. The purpose of the present study was to compare children receiving anti-TNF agents to children receiving standard treatment in regards to pain experience on a daily basis for 2 weeks, disease activity, and functional disability. We hypothesized that the children in the anti-TNF agent group, because of the effective treatment, would experience less pain, lower level of functional disability, and less disease activity compared to a standard treatment group.

Our clinic has experience with children who respond well to anti-TNF treatment in regards to measurable disease activity but still report frequent and significant pain. Thus, we also explored differences in the use of pain-coping and pain-specific beliefs in a subgroup of children with pain treated with anti-TNF agents compared to pain-free children in the treatment group. Based on previous research, we hypothesized that the children experiencing pain would express more maladaptive pain-coping strategies and pain-specific beliefs.

## Methods

### Participants

Children treated at the Pediatric Rheumatology Clinic, Aarhus University Hospital, and one of their parents were invited to participate. Inclusion criteria were a confirmed JIA diagnosis according to ILAR criteria [27], age 8–17 years, lack of comorbidity with other chronic diseases, and ability to speak fluent Danish.

#### Standard treatment group

During the period from September 2002 to February 2003, all 67 children who met the criteria for inclusion in the standard treatment group were invited to participate in the study. Sixty-four children, each with one of their parents, agreed to participate in the study. Eighteen of the children received methotrexate (36%) (Table 1). Four children who received anti-TNF agents in the period following inclusion of the standard treatment group were excluded from the standard treatment group. When data was collected for the anti-TNF agent group these four children completed the measures again and were included in the anti-TNF group. Ten children had incomplete diaries and were excluded from the analysis. The final sample consisted of 50 children (response rate = 75%). Results based on the data from this sample were published previously [21,23].

**Table 1:** Demographic characteristics of the treatment groups

	**Standard treatment group (n = 50)**	**Anti-TNF group (n = 41)**	**Group differences**	**p-values**
Age (year): mean (S.D.)	11.8 (2.0)	13.8 (2.4)	Z = −3.79	<0.001
Gender (male)	9 (18.0%)	12 (29.3%)	χ^2^(1) = 1.04	0.31
JIA subtype:			χ^2^(6) = 13.0	0.04
Systemic arthritis	5 (10.0%)	9 (22.0%)		
Oligoarthritis persistent	11 (22.0%)	1 (2.4%)		
Oligoarthritis extended	9 (18.0%)	9 (22.0%)		
Polyarthritis (RF-negative)	22 (44.0%)	17 (41.5%)		
Polyarthritis (RF-positive)	0 (0.0%)	1 (2.4%)		
Psoriatic arthritis	1 (2.0%)	2 (4.9%)		
Enthesitis-related arthritis	0 (0.0%)	2 (4.9%)		
Data on subtype n.a.	2 (4.0%)			
Disease duration (year): mean (S.D.)^a^	5.2 (3.6)	8.0 (3.6)	Z = −3.23	0.001
Current use of medication:				
NSAID use:	41 (82.0%)	17 (41.5%)	χ^2^(1) = 14.31	<0.001
Use of other analgesics:	10 (20.0%)	15 (36.6%)	χ^2^(1) = 2.33	0.13
Methotrexate use:	18 (36.0%)	17 (41.5%)	χ^2^(1) = 0.10	0.75
Anti-TNF agents use:				
Etanercept		31 (75.6%)		
Adalimumbab		3 (7.3%)		
Infliximab		3 (7.3%)		
Treatment time with anti-TNF agents (year)		2.4 (1.8)		

^a^Disease duration was defined as the time from the onset of the symptoms to the participation in the study.

#### Anti-TNF group

An additional criterion for inclusion in the anti-TNF agent group was current or previous treatment with anti-TNF agents. During the period from September 2007 to February 2008 all 51 children who met the criteria for inclusion in the anti-TNF agent group were invited to participate in the study. Fifty children, each with one of their parents, agreed to participate in the study. Nine children had incomplete diaries and were excluded from the analysis. The final sample consisted of 41 children (response rate = 80%). Thirty-one children were treated with etanercept, 3 with adalimumab, and 3 with infliximab. Four children had ended treatment with anti-TNF agents at the time of inclusion (1 was treated with etanercept, 2 with adalimumab, and 1 with infliximab; mean time since ending treatment = 1.6 years, S.D. =1.5 years).

### Procedure

Parents and children were recruited during routine visits at the clinic. Data were collected from one parent accompanying the child to the clinic. If two parents accompanied the child preference for participation was given to the child’s primary caregiver. The child and parent completed the questionnaires separately at the clinic. Before completing the questionnaires each child was examined by a pediatric rheumatologist who registered the disease activity. The children completed a pain diary twice a day during the 2 weeks following the completion of the questionnaires. The study was approved by the Ethical Review Board, Central Denmark Region, Denmark and informed consent was obtained from the parents.

### Measures

Both the children and their parents completed several questionnaires. Based on this study’s aims and hypotheses, the following scales were included in the analyses:

#### Pain diary

The pain diary measuring the children’s pain experience consisted of the Revised Faces Pain Scale (FPS-R) [28]. The FPS-R contains six faces with anchors at the two ends, representing a scale from 0,“no pain” to 5, “worst pain”. The children were instructed to measure their current pain intensity every morning and evening over the following 2 weeks. The parents were asked to help the child remember the procedure, but to avoid influencing the child’s pain assessment. The FPS-R has been recommended for research use [29].

#### Childhood health assessment questionnaire

Parents assessed the child’s level of functional disability with the Childhood Health Assessment Questionnaire (CHAQ) [30]. The CHAQ consists of 30 items and assesses the children’s performance over the past week on a 3-point Likert scale in eight areas of daily living (i.e. dressing and personal care, rising, eating, walking, hygiene, reach, grip, and activities). The CHAQ disability index was calculated by averaging the 30 items (range 0 to 3). A higher disability index indicated greater functional impairment. Chronbach's alpha was acceptable (=0.95).

#### Pain coping questionnaire

Four subscales were used from the Danish version of the Pain Coping Questionnaire (PCQ) [31]: behavioral distraction, cognitive distraction, positive self-statements, and catastrophizing. The four subscales consist of 19 items. On a 5-point Likert scale, the children indicated how often they used the strategies to cope with pain. The Cronbach’s alpha internal consistency reliability coefficients for each of the subscales were in an acceptable range (0.73-0.86).

#### Survey of pain attitudes

Three subscales were used from a revised version of the Survey of Pain Attitudes (SOPA) [32] (SOPA children’s version): control (belief in one’s personal control over pain), disability (belief in oneself as unable to function because of pain), and harm (belief that pain signifies damage and that exercise and activity should be restricted). The three subscales consisted of 26 items.

On a 4-point Likert scale, the children indicated how much they agreed with statements about their pain problem.

Cronbach’s alpha internal consistency reliability coefficients for the subscales of control and disability were acceptable (Cronbach’s alpha = 0.60-0.74). The Cronbach’s alpha was 0.57 for the harm subscale, which was less than 0.60 and therefore not considered acceptable. Therefore, one item (“Pain does not necessarily mean that my body is being harmed”) was omitted from the subscale because of weak correlation with the other items in the subscales. The Cronbach’s alpha of the subscales were then recalculated and considered acceptable (Cronbach’s alpha = 0.65).

#### Disease activity

A composite arthritis activity score as described previously [21] was calculated based on data registered by the physician as the sum of the active joint score (zero active joints = 0; 1–2 active joints = 1; 3–4 active joints = 2, >4 active joints = 3), morning stiffness (<15 minutes = 0; 15–30 minutes = 1, 30–60 minutes = 2; >60 minutes = 3), and erythrocyte sedimentation rate (<15 mm/hour = 0; 15–25 mm/hour = 1; 25–40 mm/hour = 2; >40 mm/hour = 3).

### Statistical analysis

All statistical analyses were performed using IBM SPSS version 20.0 for Windows (IBM® SPSS®, IBM Corp., Armonk, New York). The alpha level was set at 0.05 for all analyses. Mean substitution of the missing items was performed for the PCQ, and SOPA-C subscales. Number of missing on the PCQ subscales were between 0.4% (cognitive distraction subscale and positive self-statements subscale) and 1.1% (behavioral distraction), and for the SOPA subscales the number of missing were between 2.0% (control subscale) and 3.1% (disability subscale). The Kolmogorov-Smirnov test indicated that the CHAQ, FPS-R, and disease activity score was non-normally distributed and transformation was not possible. Differences between the groups were examined using the chi-square test for categorical variables and Mann–Whitney U Test for continuous variables. Effect sizes were calculated as *r* for continues variables and Phi for categorical variables [33]. Because of the effective treatment in the anti-TNF group we expected more children in the anti-TNF group to be pain-free. The group differences regarding pain intensity and functional disability were analyzed both with and without the pain-free children to investigate the pain intensity and functional disability level in the children, who experienced pain.

Differences between the treatment groups regarding socio-demographic and disease-related variables were analyzed. Variables with significant group differences were adjusted for in a regression modeling and entered as one block in the analyses. Because of the skewed distribution of the pain intensity score, the score was transformed into two sets of categorical variables. The first variables were defined as children reporting no pain at all in the pain diary or children reporting pain. The second variables were defined as either pain every day in the pain diary or pain less than every day.

## Results

The demographic characteristics of the two groups are provided in Table 1 including statistically differences between the treatment groups. A relatively high number of children with systemic arthritis received anti-TNF treatment. All children in this group had active arthritis and were without systemic symptoms.

### Comparison of pain and health status of the treatment groups

When analyzing all the participating children, children treated with anti-TNF agents experienced the same pain intensity and functional disability as children undergoing standard treatment. This is shown in Table 2. However, disease activity was significantly lower in the anti-TNF group compared to standard treatment with a large effect size. Significant more children in the anti-TNF group reported no pain at all and this result showed a moderate effect size. No differences were found regarding the number of children with daily pain.

**Table 2:** Differences in pain and health status of all participating children

	**Standard treatment group (n = 50)**		**Anti-TNF agents group (n = 41)**		**Group-differences**		
	**Mean (S.D.)**	**Median**	**Mean (S.D.)**	**Median**	**Mann–Whitney U-test**	**p-values**	**Effect sizes (*r)***
**Arthritis activity: Composite disease activity score**	2.8 (2.5)	2.00	0.65 (1.00)	0.00	Z = −4.51	<0.001	0.47
**Functional disability: CHAQ dis. index**	0.25 (0.37)	0.10	0.30 (0.32)	0.10	Z = −0.01	0.99	0.001
**Pain intensity: Mean pain diary score**	0.86 (0.88)	0.60	1.03 (1.19)	0.69	Z = −0.49	0.62	0.05
	**Number (%)**		**Number (%)**		**Chi**^**2**^**-test**	**p-values**	**Effect sizes (phi)**
**Patients with no pain reported in the pain diary**	1 (2.0%)		13 (31.7%)		χ^2^(1) = 15.27	<0.001	0.41
**Patients with daily pain reported in the pain diary**	12 (24.0%)		12 (29.3%)		χ^2^(1) = 0.32	0.64	0.06

When excluding children with no pain at all reported in the diary from the analyses significant differences were found between the standard treatment group and anti-TNF group regarding disease activity and pain intensity. As shown in Table 3 children in the anti-TNF group had significant lower disease activity and this difference showed a large effect size. However, children in the anti-TNF group reported significant more pain and the effect size of the difference was moderate.

**Table 3:** Differences in pain and health status of children with pain reported in the pain diary

	**Standard treatment group (n = 49)**		**Anti-TNF agents group (n = 28)**		**Group-differences**		
	**Mean (S.D.)**	**Median**	**Mean (S.D.)**	**Median**	**Mann–Whitney U-test**	**p-values**	**Effect sizes (*r)***
**Arthritis activity: composite disease activity score**	2.90 (2.53)	2.00	0.68 (0.99)	0.00	Z = −3.95	<0.001	0.45
**Functional disability: CHAQ dis. index**	0.26 (0.38)	0.10	0.27 (0.31)	0.18	Z = −0.95	0.34	0.04
**Pain intensity: mean pain diary score**	0.88 (0.88)	0.61	1.50 (1.16)	1.34	Z = −2.57	0.01	0.29

In logistic regression analyses the following variables were entered as predictor variables, because of significant treatment group differences in the previous analyses: treatment group, age, disease duration, subtype, use of NSAIDs, and disease activity. For the categorical variable of children reporting no pain in the diary, the model containing the predictor variables was significant (χ^*2*^(11) = 25.84, *p* = 0.007). The model as a whole explained between 27% (Cox & Snell R square) and 48% (Nagelkerke R square) of variance and correctly classified 87% of cases. As shown in Table 4 none of the predictor variables made a unique significant contribution. For the categorical variable of the patient reporting daily pain, a test of the full model against a constant-only model did not reach a significant level, indicating that the predictors as a set could not reliably be distinguished between the categories (χ^*2*^(11) = 10.78, *p* = 0.46). The Cox & Snell R square of 12% and Nagelkerke R square of 18% indicated a weak relationship between prediction and grouping. Overall prediction success was 77%, and as shown in Table 4 none of the variables made a significant contribution to prediction.

**Table 4:** Overview predictor variables in the logistic analyses

**Dependent variable: children reporting no pain in the diary or children reporting pain**						
**Predictor**	**β**	**S.E.**	**Wald’s χ**^**2**^	**df**	***p***	**e**^**β **^**(odds ratio)**
**Constant**	0.37	2.50	0.02	1	0.88	1.45
**Treatment group**	−2.22	1.35	2.71	1	0.1	0.11
**Age**	−0.01	0.02	0.06	1	0.80	1.00
**Disease duration**	−0.01	0.10	0.06	1	0.81	1.00
**Use of NSAID**	−0.71	0.83	0.74	1	0.39	0.49
**Disease activity**	−0.84	0.67	1.58	1	0.21	0.43
**Subtype**			0.10	6	1.00	
**Dependent variable: children reporting daily pain in the diary or not**						
**Predictor**	**β**	**S.E.**	**Wald’s χ2**	**df**	***p***	**e**^**β **^**(odds ratio)**
**Constant**	−2.664	2.065	1.663	1	.197	.070
**Treatment group**	−1.436	.806	3.177	1	.075	.238
**Age**	.002	.011	.030	1	.863	1.002
**Disease duration**	-.003	.007	.262	1	.609	.997
**Use of NSAID**	1.157	.698	2.744	1	.098	3.180
**Disease activity**	.114	.146	.606	1	.436	1.120
**Subtype**			2.411	6	.878	

### Comparing children with pain and pain-free children in the anti-TNF agent group

A subgroup of children in the anti-TNF agent group (n = 28) who reported pain in the pain diary was compared with pain-free children in the anti-TNF agent group (n = 13). No differences were found between the two pain subgroups in regards to disease activity and age. The female: male gender ratio in the pain-free subgroup was 1.17:1, compared to 3.67:1 for the subgroup with pain.

As shown in Table 5 the subgroup with pain tended to use the pain-coping strategy of catastrophizing more than the pain-free children in the treatment group. This difference was significant and with a moderate effect size. The pain subgroup was also significantly more likely to believe that pain signifies damage, and that they were unable to function because of pain. These differences showed moderate effect sizes.

**Table 5:** Comparison of pain-coping and pain-specific beliefs in children with and without pain in anti-TNF treatment

**Anti-TNF agents group**							
	**Subgroup with pain (n = 28)**		**Pain-free subgroup (n = 13)**		**Group differences**		
	**Mean (S.D.)**	**Median**	**Mean (S.D.)**	**Median**	**Mann–Whitney U Test**	**p-values**	**Effect sizes ( *r *)**
**Pain-coping strategies**							
Behavioural distraction	2.94 (0.86)	2.70	3.02 (1.01)	3.40	Z = −0.62	0.54	0.10
Cognitive distraction	3.27 (0.93)	3.30	2.88 (1.03)	3.00	Z = −1.04	0.30	0.16
Positive self statements	2.91 (0.96)	3.10	2.83 (1.19)	2.60	Z = −0.07	0.94	0.01
Catastrophizing	1.99 (0.93)	1.63	1.43 (0.70)	1.20	Z = −2.03	0.04	0.32
**Pain-beliefs**							
Control	2.84 (0.57)	2.89	2.83 (0.71)	3.00	Z = −0.12	0.91	0.02
Disability	2.69 (0.84)	2.56	1.96 (0.56)	1.89	Z = −2.68	0.007	0.42
Harm	3.38 (0.74)	3.50	2.81 (0.55)	2.83	Z = −2.55	0.01	0.40

## Discussion

On average, the children treated with anti-TNF agents in this 2-weeks diary study reported low pain, but contrary to our hypotheses no differences were found between the groups in terms of functional status and the average pain experience over 2 weeks. The socio-demographic and disease-related variables did not account for the difference or lack of difference between the pain variables. Our results indicate that the majority of the children treated with anti-TNF agents did respond well to the treatment regarding disease activity, which was significantly lower compared to children in standard treatment, and a higher amount of children in the anti-TNF group (almost 1/3) had no pain at all in 2 weeks. However 29% of the children treated with anti-TNF agents reported pain on a daily basis despite treatment with anti-TNF agents. To the best of our knowledge no previous studies have assessed pain frequency solely in children treated with anti-TNF agents. In two diary studies with children with arthritis 17% [34] and 39% [1] reported pain on a daily basis. However, besides from only including a subsample of children treated with anti-TNF agents (about a quarter of the children were treated with anti-TNF agents), the pain assessment method was different from ours in the first study, and in the second study only children diagnosed with polyarticular arthritis were included, which makes meaningful comparisons between the studies difficult.

When excluding the pain-free children from the analyses the difference between the treatment groups regarding disease activity maintained, but despite the lower disease activity, the children in the anti-TNF agent group experienced significant higher pain intensity. The lack of association between disease activity and the level of experienced pain has been found in other studies as well. In a recent study with 2795 adults with arthritis, 75%-82% reported a pain level of moderate to severe, though they rated their arthritis as somewhat to completely controlled [35]. In our previous study no changes were detected in the level of pain experience in JIA children over a 2-years period even though the disease activity was significantly reduced over the 2 years [23]. In other studies however a strong association between disease activity and pain intensity has been demonstrated [16,17,36,37]. However, with the use of multivariate statistical methods, the severity of the disease has been shown to predict only a small to medium part of the variance in pain intensity reported by the children [18-20].

There are several possible reasons why pain persists in the absence of significant disease activity. Central sensitization causing hyperalgesia may be a biological factor affecting the pain experience in children with JIA. We and others have found significantly reduced pain threshold and pain tolerance in children with arthritis compared with healthy schoolchildren [22,38-40], which supports the theory that chronic pain conditions may cause central sensitization [41,42]. The amount of pain experienced by a child with JIA may independently of the inflammatory disease be modulated by psychological and social factors as well [43,44]. Studies have found that parental factors as the parent’s pain history [45], personality profile [46], and family function [47,48] may have an impact on the child’s pain experience. Beside’s pain coping and pain-specific beliefs the child’s self-efficacy for managing the disease [49-51] and emotional wellbeing [1,47,52,53] has been related to the child’s pain experience as well.

Our second hypothesis was confirmed: children treated with anti-TNF agents, who reported pain in the diary tended to use the pain-coping strategy of catastrophizing more, they were significantly more likely to believe that pain signifies damage, and that they were unable to function because of pain, than the pain-free children in the treatment group. The proportion of girls in the pain subgroup was larger than in the pain-free subgroup. Girls tend to experience more pain [54,55] and more frequently use the pain-coping strategy of catastrophizing than boys [55,56]. Differences in the gender ratio could have an impact on the difference between the subgroups. However, the result is similar to our previous findings with children experiencing high pain despite low disease activity [21,22]. Based on the design of this and our previous studies a causal relationship between pain cognitions and pain experience cannot be inferred. In a study with adult chronic pain patients, changes in the pain beliefs of control, disability, and harm, pain catastrophizing, and self-efficacy for managing pain mediated the effects of cognitive behavioral therapy (CBT) on pain, and thus indicates that pain cognitions are influencing pain experience [57]. There is a need of further intervention studies to test whether these mechanism apply for children with JIA.

To the best of our knowledge, this study is the first to investigate daily pain experience in children treated with anti-TNF agents, and to compare the pain scores with pain reported by children receiving standard treatment. However, some limitations exist in the interpretation of the results of the present study. First, data from the treatment groups was collected on two occasions, and we are aware that the children are not clinically comparable. The children included in the anti-TNF group have most often started anti-TNF treatment because of inefficacy or intolerance to the DMARD therapy. However, the attitude towards and use of analgesia and intra-articular steroid injections did not change in the clinic in the period between the data collections. Second, the disease activity index has not been validated in a larger patient population. However, the disease activity score used in this publication was also used in our previous, related publications [21,23]. We therefore found it most reasonable to use this scoring method in the present paper since the cohort of children receiving standard treatment was ascertained from the previous investigations. The currently used activity score Juvenile Arthritis Disease Activity Score (JADAS) [58], now being an accepted method of evaluating disease activity for children with JIA, was not implemented when our study was started. Third, because of the distribution of the mean pain intensity score we had to create categorical variables from the continuous scale to control for treatment group differences regarding pain in regression analyses. Fourth, the cross-sectional design of the study does not allow the inference of causal relationships.

## Conclusions

In summary, significant conclusions can be drawn from our results. A large proportion of children treated with anti-TNF agents respond well to the treatment both in regards to pain and disease activity. But for a subgroup of these children pain is still a problem even though the disease is well-controlled. Further studies to investigate the characteristics of this subgroup are needed. More knowledge about predictors of ongoing pain despite reduced disease activity may help detecting the children early in the treatment course and provide these children with appropriate interventions. Psychological interventions may contribute to diminish pain complaints and improve quality of life in children with JIA who are continuing to report pain despite appropriate medical treatment.

## Competing interests

The authors declare that they have no competing interests.

## Authors’ contributions

JJL is the corresponding author and has substantially contributed to the study conception and design, in acquisition, analysis and interpretation of data and in drafting of the manuscript. MT substantially participated in the study conception and design, in the analysis and interpretation of data and in editing of the manuscript. TH has substantially contributed in the study conception and design, in acquisition and the interpretation of data, and in editing of the manuscript. All authors read and approved the final manuscript.
